# The Association of Anti-Sm with Osteopontin Related to Cognitive Impairment in a Pristane-Induced Lupus BALB/c Mice Model

**DOI:** 10.3390/ijms252313080

**Published:** 2024-12-05

**Authors:** Daniel González-Inostroz, Flavio Sandoval-García, Fernanda-Isadora Corona-Meraz, Mónica Vázquez Del Mercado, Jorge Guzmán-Muñiz, Milton Omar Guzmán-Ornelas, Rolando Castañeda-Arellano, Jacinto Bañuelos-Pineda, Miguel Peña-Nava, Beatriz-Teresita Martín-Márquez

**Affiliations:** 1 Departamento de Biología Molecular y Genómica, Instituto de Investigación en Reumatología y del Sistema Músculo Esquelético, Centro Universitario de Ciencias de la Salud, Universidad de Guadalajara, Guadalajara 44340, Jalisco, Mexico; dgi-17@hotmail.com (D.G.-I.); dravme@hotmail.com (M.V.D.M.); miguel.pena4206@alumnos.udg.mx (M.P.-N.); 2 Departamento de Neurociencias, Centro Universitario de Ciencias de la Salud, Universidad de Guadalajara, Guadalajara 44340, Jalisco, Mexico; 3 Cuerpo Académico UDG-CA-703, Inmunología y Reumatología, Centro Universitario de Ciencias de la Salud, Universidad de Guadalajara, Guadalajara 44340, Jalisco, Mexico; 4 Departamento de Ciencias Biomédicas, Centro de Investigación Multidisciplinario en Salud, Centro Universitario de Tonalá, Universidad de Guadalajara, Guadalajara 45425, Jalisco, Mexico; mariaf.corona@academicos.udg.mx (F.-I.C.-M.); milton.guzman@academicos.udg.mx (M.O.G.-O.); rolando.castaneda@academicos.udg.mx (R.C.-A.); 5 Servicio de Reumatología, División Medicina Interna, Hospital Civil de Guadalajara “Dr. Juan I. Menchaca”, Guadalajara 44340, Jalisco, Mexico; 6 Laboratorio de Neurociencias, Facultad de Psicología, Universidad de Colima, Colima 28040, Colima, Mexico; guzman72@ucol.mx; 7 Laboratorio de Morfofisiología, Centro Universitario de Ciencias Biológicas y Agropecuarias, Universidad de Guadalajara, Zapopan 45510, Jalisco, Mexico; jacinto.banuelos@academicos.udg.mx

**Keywords:** systemic lupus erythematosus, neuropsychiatric lupus, pristane-induced lupus, osteopontin, anti-Sm antibodies, spatial learning and memory

## Abstract

The BALB/c model of pristane-induced lupus (PIL) exhibits cognitive impairment features resembling neuropsychiatric lupus (NPLSE). Osteopontin (OPN) is associated with disease activity in SLE; however, its involvement in NPLSE is not yet entirely determined. Our study aims to elucidate the contribution of full-length OPN (OPN-FL) plasma expression, OPN N-half, and *Spp1* to cognitive impairment in the PIL mice model. A total of 76 female BALB/c mice were divided into pristane (P), pristane plus lipopolysaccharide (P plus LPS) and control (C) groups. In behavioral tests, the P group showed cognitive and visuospatial memory impairment. Elevated plasma OPN FL levels were found in P compared to C groups (177.7 ± 90.1 vs. 105.9 ± 56.8 ng/mL, *p* = 0.009) and OPN N-half was different between P and C groups (673.5 ± 144.6 vs. 624.5 ± 377.7 ng/mL, *p* = 0.028) and P plus LPS and C groups (624.5 ± 377.7 vs. 381.4 ± 205.0 ng/mL, *p* = 0.001). Anti-Sm correlated with OPN-FL (r = 0.269, *p* = 0.0150). The relative expression of *Spp1* in the brain was 2.5 and 2.7-fold higher in P and P plus LPS groups, respectively. The evidence suggests that OPN is related to cognitive impairment in PIL mice and might play a relevant role in the detrimental neurological conditions of NPSLE.

## 1. Introduction

Systemic lupus erythematosus (SLE) is a heterogeneous, chronic autoimmune disease with a wide range of clinical manifestations, which can vary its prognosis [1,2]. SLE pathogenesis is related to the complex immune dysregulation driven by interactions between the immune innate and adaptive systems, a process that leads to the upregulation of cytokines such as type I interferon (IFN-type I), complement activation, immune-complex deposition, the production of autoantibodies against nuclear antigens, and inflammation and tissue damage [2,3]. One specific cellular antigen recognized by autoantibodies in SLE is the Smith, known as Sm. Anti-Sm antibodies are directed against this antigen and are considered pathognomonic of the disease [4].

SLE affects multiple organ systems, including the mucocutaneous, musculoskeletal, hematologic, kidney, and nervous systems [3]. Approximately 5–10% of SLE patients suffer from nervous system complications, termed neuropsychiatric lupus erythematosus (NPLSE). These complications manifest as central nerve system (CNS) or peripheral nerve system (PNS) involvement, which is acute or chronic, diffuse or localized. The American College of Rheumatology (ACR) proposed in 1999 a set of definitions for 19 NPSLE syndromes to homogenize the terminology for research and clinical practices; nevertheless, the prevalence of NPLSE varies widely according to different series and is estimated to be between 37 and 95% [5].

The onset of NPSLE occurs in the early active stage of the disease course; NPLSE is known as the leading cause of death in SLE patients, second only to renal involvement. In this regard, it has been estimated that the mortality rate of NPLSE patients is 11.14 times higher than that of the general population. Seizures, cerebrovascular disease, acute confusion state, cognitive dysfunction, and psychosis are the most common manifestations of central lupus [6].

Patients with NPLSE develop mild to moderate cognitive impairment, which progresses benignly; this form of presentation has been estimated to occur with a prevalence of 14–95%. On the other hand, severe cognitive impairment has been observed to develop in 3–5% of patients with NPLSE. Attention, visual and verbal memory, executive function, and psychomotor speed are these patients’ most prevalent clinical manifestations of cognitive impairment [5].

NPLSE is a frequent manifestation of SLE, and the underlying mechanism remains elusive, resulting in a lack of therapeutic targets [7]. Therefore, experimental models of lupus are considered to be an alternative for studying some clinical features that resemble human pathology, understanding immunopathogenesis, and managing diseases with the generation of novel therapeutics, which are some of the main achievements of these models. Lupus-like disease in murine models is the most extensively used representation and has been classified into two categories: spontaneous and induced. The latter is characterized by the involvement of the environment in the development of autoimmunity [8].

The pristane-induced lupus (PIL) mice model is considered an example of how environmental factors can predispose an individual to SLE development. This model is associated with IFN-I overproduction and the expression of interferon-stimulated genes known as “IFN signature”, closely related to disease activity, lupus nephritis, and autoantibody production [9]. Regarding NPLSE alterations, PIL mice exhibit a series of characteristic behavioral deficits and pathological changes in the brain, which make them suitable for investigating immunopathogenesis and evaluating potential therapeutic targets [10].

Osteopontin (OPN) is a glycosylated phosphoprotein involved in bone homeostasis, healing, cell adhesion, angiogenesis, immune response, and tissue remodeling. OPN is widely distributed in human tissues where it is abundantly expressed in the bone matrix of osteoclasts and osteoblasts, as well as in the kidneys, epithelial cells of the gastrointestinal tract, the gallbladder, the pancreas, the urinary and reproductive tracts, among others, and by cells of the innate and adaptive immune system.

In humans, the OPN-encoding gene is identified as SPP1 (secreted phosphoprotein-1), whereas in rodents, it is known as *Spp1* [11]. OPN activity is regulated by thrombin, which converts OPN full-length (OPN FL) into OPN N-half [12].

In physiological conditions, OPN is expressed in low concentrations in the CNS [13]. However, there is evidence for the involvement of OPN in neurodegenerative diseases such as Parkinson’s disease, Lewy body disease, and Alzheimer’s disease through its action as a trigger of inflammation [14]. In Parkinson’s patients, elevated levels of OPN in the serum and cerebrospinal fluid (CSF) have been found to correlate with dementia and the severe motor phenotype, respectively [15]. On the other hand, it has been documented that hippocampal neurons from Alzheimer’s patients and their respective experimental models overexpress OPN and are surrounded by amyloid plaques [16].

In SLE, OPN may play an essential role in the pathophysiology because it has been found in increased concentrations in serum and urine samples correlating with disease activity [17]. Although OPN has been found in CSF in NPLSE patients and proposed as a novel diagnostic marker [17], it is still unknown whether it is related to the clinical and behavioral manifestations of NPSLE. Therefore, this study aims to elucidate the contribution of plasma OPN FL, OPN N-half, and *Spp1* brain expression in visuospatial learning and memory alterations evaluated with the Barnes maze (BM) behavioral test in a PIL mice model.

## 2. Results

### 2.1. Deficiencies in the Memory Consolidation Process in the PIL Mice Model

**Figure 1:**
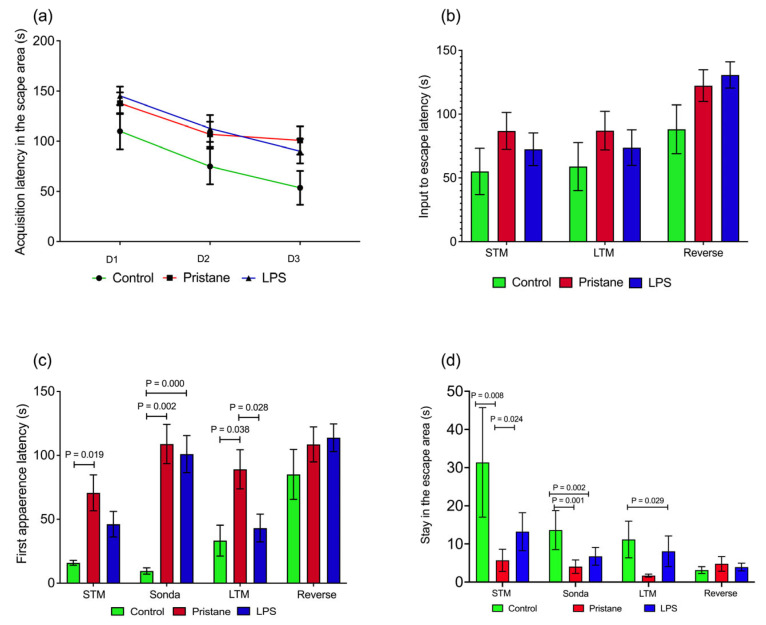
Visuospatial learning and memory latencies were assessed on BM tests. (**a**) Escape latency in the acquisition phase; (**b**) escape latency in the BM trials; (**c**) first appearance latency in the escape area; and (**d**) permanency in the escape area. Data are presented as the means ± standard error of the mean (SEM). *p* values from the Kruskal–Wallis test. Abbreviatures: s = seconds; d = day; STM = short-term memory; LTM = long-term memory; LPS = lipopolysaccharide.

Our study aimed to evaluate the experimental groups’ hippocampal-dependent spatial and learning memory using the BM task, including evaluations by a researcher and use of the EthoVision^TM^ software 3.1.16 software (Noldus Information Technology). At the end of the acquisition phase (d5), the pristane (P) and pristane plus lipopolysaccharide (P plus LPS) groups showed prolonged latency when entering the escape target compared to the control (C) group. Nevertheless, no differences were obtained; there was a tendency for the C group to decrease the time of entry to the escape hole (Figure 1a). Due to the above, we observed a continuous and efficient search attitude in C compared to P and P plus LPS groups, in which higher latencies were observed, specifically in the P-treated group.

Concerning memory consolidation tests (executive and visuospatial memory) evaluated in the BM during short-term memory (STM), long-term memory (LTM), and reverse tests, we noted that the P and P plus LPS groups tended to extend the latency times of entry to the escape orifice when exposed to the cognitive paradigm (Figure 1b).

On the other hand, when the first appearance latency in the escape hole was assessed, we observed that group C was more efficient than the P and P plus LPS-treated mice in the cognitive demand tests, in which we noted the following differences: in STM tests, the C group showed differences between the P-treated groups (15.9 ± 1.9 vs. 70.7 ± 14.0 s; *p* = 0.019); in sonda, the C group displayed differences between the P and P plus LPS groups (9.6 ± 2.4 vs. 108.9 ± 15.2 s; *p* = 0.002 and 9.6 ± 2.4 vs. 101.0 ± 14.4 s; *p* = 0.000, respectively), and in LTM tests, the C group showed differences between the P and P plus LPS groups (33.3 ± 12.0 vs. 89.1 ± 15.2 s; *p* = 0.038 and 89.1 ± 15.2 vs. 43.2 ± 10.8 s; *p* = 0.028, respectively). The first appearance of latency in the reverse test in the study groups revealed a decreasing trend for group C. This indicates that visuospatial memory improved searchability when switching from the escape hole to the opposite side (Figure 1c).

Regarding the permanency in the escape area test, in STM, sonda, and LTM tests, the permanence of the C group in the quadrant and escape hole was higher compared to the P and P plus P groups, and are evidenced by the results below: STM, C between P and P plus LPS (31.3 ± 14.3 vs. 5.7 ± 2.9 s; *p* = 0.008 and 31.3 ± 14.3 vs. 13.2 ± 4.9 s; *p* = 0.024, respectively); sonda, C between P and P plus P (13.6 ± 5.1 vs. 4.0 ± 1.7 s; *p* = 0.001 and 13.6 ± 5.1 vs. 6.7 ± 2.3 s; *p* = 0.002, respectively); and LTM, C between P plus LPS (11.1 ± 4.8 vs. 8.0 ± 4.0 s; *p* = 0.029). In contrast, in the reverse test, there were no differences (Figure 1d). 

**Figure 2:**
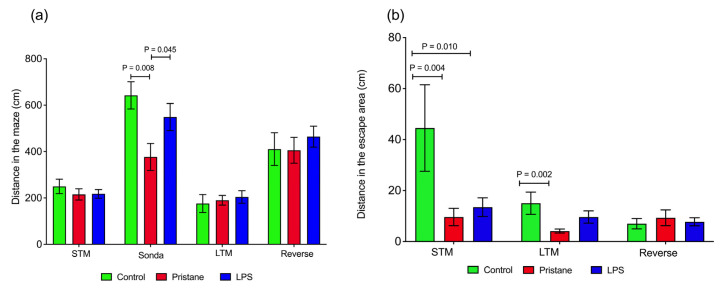
Visuospatial learning and memory distances were assessed on BM tests. (**a**) The distance in the maze; (**b**) the distance in the escape area. Data are presented as the means ± SEM. *p* values from the Kruskal–Wallis test. Abbreviatures: cm = centimeters; STM = short-term memory; LTM = long-term memory; LPS = lipopolysaccharide.

Concerning the evaluations of the traveled distances assessed, we evaluated the following parameters: the distance traveled in the maze, and the distance traveled to the escape hole. Regarding the first evaluation, which measured the total distance traveled on the platform, we only observed differences in the sonda, in which the C group presented differences between the P group (642.3 ± 57.9 vs. 376.7 ± 58.0 cm; *p* = 0.008) and the P group presented differences between the P plus LPS group (376.7 ± 58.0 vs. 549.0 ± 58.3 cm; *p* = 0.045). Data are presented in Figure 2a.

Regarding the distance parameter in the escape hole area, differences were obtained in the STM test between the C group and the P-treated groups (44.5 ± 16.9 vs. 9.6 ± 3.3 cm; *p* = 0.004 and 44.5 ± 16.9 vs. 13.4 ± 3.6 cm; *p* = 0.010, respectively) as well as the LTM test between the C and P groups (15.0 ± 4.3 vs. 4.1 ± 0.7 cm; *p* = 0.002). In Figure 2b, the data are presented.

### 2.2. OPN FL and OPN N-Half Plasma Levels Were Higher in the P Group

**Figure 3:**
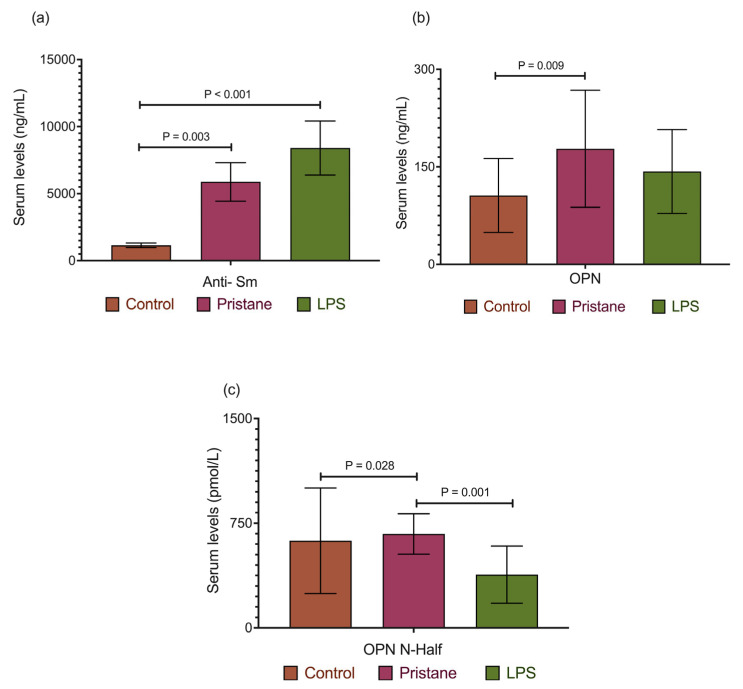
Anti-Sm antibodies, OPN FL, and OPN N-half plasma levels in the PIL model. (**a**) Anti-Sm antibodies plasma levels, (**b**) OPN FL plasma levels, and (**c**) OPN N-half plasma levels. Data are presented as the means ± SEM—*p* values from an ANOVA test.

Once the BM tests were performed, a blood sample was taken to determine the levels of plasma-circulating soluble molecules. We noticed that 75% of mice in the experimental groups (P and P plus LPS) were positive for plasma anti-Sm antibodies. Significative differences were found between the P and C groups (5877.3 ± 1438.2 vs. 1151.1 ± 168.7 ng/mL, *p* = 0.003) and the P plus LPS vs. C groups (8403.8 ± 2013.6 ng/mL vs. 1151.1 ± 168.7; *p* < 0.001), which are shown in Figure 3a.

The plasma OPN FL levels were high in the P and P plus LPS groups; however, differences were only observed between the P and C groups (177.7 ± 90.1 vs. 105.9 ± 56.8 ng/mL, *p* = 0.009). Regarding OPN N-half, differences were observed between the P and C groups (673.5 ± 144.6 vs. 624.5 ± 377.7 ng/mL, *p* = 0.028) and the P plus LPS and P-treated groups (624.5 ± 377.7 vs. 381.4 ± 205.0 ng/mL, *p* = 0.001). Data are noted in Figure 3b,c, respectively.

### 2.3. OPN FL and OPN N-Half Plasma Levels Were Higher for Anti-Sm Antibody-Positive Mice

**Figure 4:**
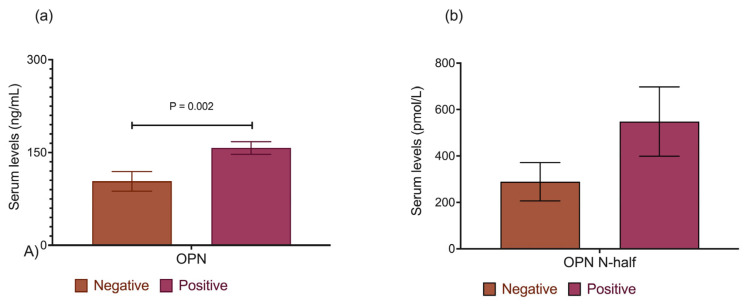
OPN FL and OPN N-half plasma levels among anti-Sm antibodies positive mice. (**a**) OPN FL plasma levels among anti-Sm antibody-negative and positive mice, (**b**) OPN FL plasma levels among anti-Sm antibodies negative and positive mice. Data are presented as the means ± SEM. *p* values from the ANOVA test.

An analysis performed to compare OPN FL and OPN N-half plasma levels with anti-Sm levels revealed that they were found in higher concentrations in the mice positive to anti-Sm, obtaining significance with OPN FL (103.4 ± 15.7 vs. 157.3 ± 10.2, *p* = 0.002). The plasma levels of OPN FL and OPN N-half in the anti-Sm-positive mice are shown in Figure 4.

### 2.4. Relationship of Plasma Anti-Sm Antibodies with OPN FL

**Figure 5:**
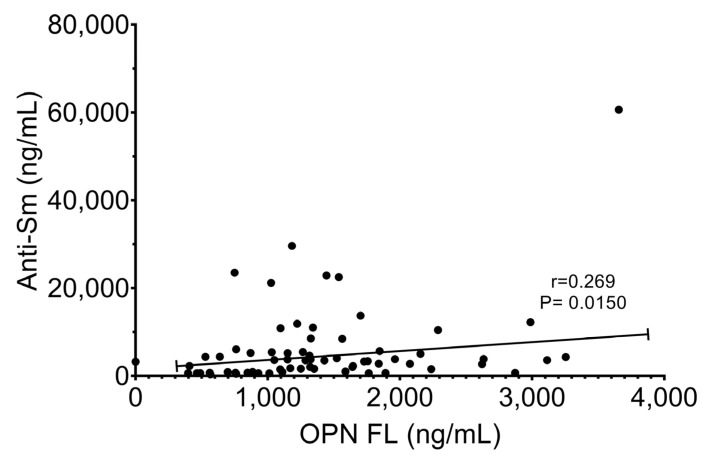
Relationship of plasma anti-Sm with OPN FL. Spearman’s correlation test was carried out.

Once the differences between the experimental groups’ anti-Sm and OPN FL levels were observed, we performed a correlation analysis. As shown in Figure 5, anti-Sm correlates positively with OPN FL (r = 0.269, *p* = 0.0150).

### 2.5. Spp1 Is Overexpressed in Brain Tissue in PIL Mice Groups

**Figure 6:**
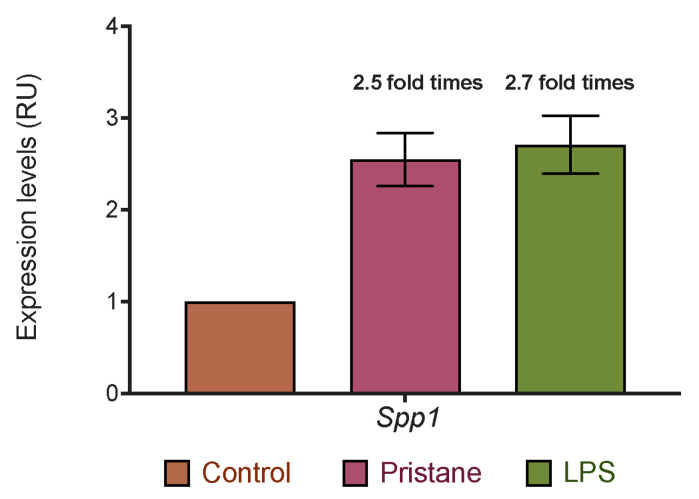
Relative *Spp1* expression in brain tissue in PIL mice. Data are presented as the means ± SEM. *p* values from the Kruskal–Wallis test.

The *Spp1* gene in rodents encodes OPN, and an analysis of its expression in brain tissue was performed in the PIL model. The relative expression of *Spp1* in the CNS was found to be 2.5 and 2.7-fold times more abundant in the P and P plus LPS groups, respectively, compared with the C groups. These results are shown in Figure 6.

## 3. Discussion

### 3.1. PIL Mouse Model for the Study of Neuropsychiatric Alterations in SLE

Diagnosing neuropsychiatric alterations in SLE patients is a complex task, with multiple factors at play. Clinicians play a crucial role in this process, considering the type and severity of symptoms, the patient’s demographic profile, and the various definitions used. Their efforts, combined with recent advancements in early diagnosis and patient evaluation, have led to improved survival rates. However, NPLSE remains a significant challenge, ranking second only to lupus nephritis in terms of morbidity and mortality [5].

Multiple efforts continue to study in meticulous detail the mechanisms by which cells, inflammatory molecules, and systemically produced autoantibodies cross the blood–brain barrier (BBB) and promote neuroinflammation. This careful and thorough approach is necessary as the study of the CNS is limited and highly invasive. Researchers have developed animal models that mimic human pathophysiology to overcome these challenges, thereby elucidating disease mechanisms. The variety of data obtained in the present investigation has led us to subdivide the analysis of the results into the following sections.

### 3.2. Cognitive-Behavioral Alterations in PIL Mouse Model

The PIL mice model in female BALB/c mice manifests SLE’s main features: immune system dysregulation, the induction of autoimmunity, and glomerulonephritis triggered by hydrocarbon oil exposure [9]. Regarding neuropsychiatric manifestations, the PIL model exhibits pathological and behavioral characteristics that make it suitable for studying NPLSE alterations [10]. In previous results published by our research team, we demonstrated that P and P plus LPS-treated groups exhibit memory and learning disturbances associated with dysregulation of the *NR2A* and *NR2B* hippocampal subunits [18]. On the other hand, Yun and colleagues showed that the PIL model manifests olfactory malfunction accompanied by an anxiety- and depression-like phenotype. In addition, they observed increased proinflammatory cytokines and chemokines in the brain, increased permeability of BBB, and behavioral alterations, suggesting that the PIL mice model can be used to evaluate therapeutic targets related to NPSLE [10]. Therefore, one of the main achievements in studying the PIL mice model related to neuropsychiatric alterations is the discovery of molecules that could be potential candidates for biomarkers to improve the diagnosis, prognosis, and treatment of NPSLE.

Barnes behavioral testing was used to evaluate the visuospatial executive memory processes in STM and LTM tests of cognitive demand, such as sonda and reverse tests. An interesting aspect of our research is the development of neurobiological associations during cognitively demanding tests. During the acquisition phase in the BM, continuous learning generates a more efficient cognitive process in which short-term and visuospatial memory consolidates the development of LTM. This process leads to the intriguing development of neurobiological associations due to increased dendritic spines and axonal projections [19,20].

Memory consolidation is relevant for cognitive efficiency; altering this process leads to learning difficulties during demanding tests. In our study, deficiencies in the consolidation process were evidenced in the P-treated group when a longer latency and shorter time spent in the escape area were observed. Although the P-treated group learned and identified the escape area, it showed a deficiency in visuospatial executive memory of the platform, cognitive reinforcement, and search attitude. These results corroborate our previous findings and support the data obtained by Yun et al., in which the PIL mice model shows pathological changes at the CNS level that reflect cognitive-behavioral alterations [10]. At this point, we consider mentioning the P plus LPS group, in which we observed a lower affectation evidenced in BM tests; this behavior may be related to compensatory mechanisms. It has been shown that chronic inflammatory responses may have a neuroprotective capacity mediated by the production of neurotrophins, so we infer that chronic exposure to LPS could induce molecules such as the brain-derived neurotrophic factor (BDNF) that have a preponderant role in the attenuation of cognitive dysfunction [21,22].

### 3.3. Association of Anti-Sm with OPN-FL in PIL Mice Model

OPN is a phosphorylated glycoprotein produced by various cells and involved in bone homeostasis, cell adhesion, angiogenesis, and immune response processes. Even though OPN is involved in crucial physiological processes, it has also been associated with multiple pathologies, including chronic inflammation, muscle inflammation, kidney and bone disease, cancer progression, metastasis, and cerebrovascular disease. The physiological function of OPN is regulated by proteolytic cleavage conducted by enzymes, including thrombin, which originates the OPN N-half fragment. This fragment exposes a new C-terminal sequence and allows accessibility to the RGD motif by integrins, performing a variety of features [12]. Both OPN FL and OPN N-half are known to be associated with SLE immunopathology and could be related to alterations at the CNS level in neuropsychiatric manifestations.

One of the main features of the PIL mice model that is considered to be the primary factor in the pathogenesis of SLE is autoantibody production. This includes anti-double-stranded DNA (dsDNA), anti-chromatin, anti-RNA, and anti-Sm antibodies, detectable about three to four months after lupus induction [9]. The PIL mice produce high levels of anti-Sm, comparable to those observed in humans when spontaneous autoimmune alterations occur. Thus, it is suitable for analyzing the main alterations at the level of autoantibody production observed in patients with SLE [23].

We found that 75% of P and P plus LPS-treated mice were positive for anti-Sm autoantibodies and manifested elevated plasma OPN FL levels compared to untreated mice. These results concord with SLE patients’ analyses, in whom high OPN FL plasma titers are associated with various manifestations, such as lupus nephritis (LN), joint damage, antiphospholipid syndrome, and disease activity [24,25,26,27]. Regarding neuropsychiatric alterations, higher titles of anti-Sm antibodies in CSF were associated with an acute confusional state (ACS), a severe form of NPLSE diffuse, which classifies anti-Sm as an autoantibody with possible neurotoxic activity and as a BBB disruptor [20]. We found a positive correlation between circulating anti-Sm antibodies and OPN-FL, which suggests that both may be related to CNS alterations. Thus, OPN could be considered a protein associated with the production of autoantibodies in the PIL model and possibly related to the induction of anti-Sm antibodies.

### 3.4. P-Treated Group Present Higher Titles of Plasma OPN N-Half

On the other hand, we decided to quantify plasma OPN N-half levels in the experimental groups to determine its concentration in the PIL mice model and any possible involvement in cognitive impairment. In our study, the P-treated group presented higher circulating levels of OPN N-half than the LPS-treated group. These findings agree with those of Kitagori et al., who showed that OPN N-half in urine was found in higher concentrations in patients with overt proteinuria and LN, demonstrating that the cleaved form could be considered a biomarker of disease activity [28]. Although we did not evaluate urine, we previously demonstrated that significant proteinuria (greater than 100 mg/dL) was observed in the group treated with P plus LPS. From this, we deduced that the greater amount of OPN N-half is eliminated via the urinary route, and consequently, its concentration in circulation is decreased [18].

### 3.5. The Overexpression of Spp1 in the Brain May Be Related to Cognitive Alterations in the PIL Mice Model

The upregulation of *Spp1* (a gene encoding for OPN in rodents) has been associated with autoimmunity-induced inflammation [11]. After the behavioral test, we quantified the *Spp1* expression levels in brain tissue and noted that the P and P plus LPS groups overexpressed *Spp1* at the CNS level. These findings are consistent with observations made in murine models of lupus with neuropsychiatric involvement. Nomura et al. in lupus-prone mice CNS resident cells found that *Spp1* expression is associated with a unique state of chronic low-grade inflammation in microglia termed “lupus-associated microglia”, characterized by the upregulation of IFN-responsive gene expression [29]. Moreover, Makinde and colleagues conducted studies in SLE-prone CReCOM (Caspase-8 Removed CD11c-specific Overactive MyD88) mice. They identified a class of microglia with an “NPSLE” signature that displays genes related to positive macrophage activation, including *Spp1* [30].

Therefore, *Spp1* expression in the CNS could act as an immune trigger that activates microglia toward the M1 phenotype, which has been implicated in neuroinflammation in patients with NPLSE. This discovery opens up new avenues for further research to elucidate the involvement of OPN in the activation of microglia related to neuropsychiatric pathology in SLE. The results obtained in the present study, while enlightening its association with anti-Sm antibodies and cognitive impairment at the experimental level, also underscore the need for continued exploration in this field.

### 3.6. Limitations and Perspectives

The main limitations in our study relate to verifying the expression of OPN FL and its cleaved forms in brain tissue, such as the hippocampus. Our primary objective in future trials will be to determine OPN as a molecule of interest related to neurodegeneration and cognitive-behavioral disturbances described in the PIL mice model. Furthermore, detecting OPN immunoreactivity in microglia is also a key focus, as it involves CNS detrimental effects by modulating microglia reactivity and neuroinflammation. On the other hand, determining proteinuria and OPN N-half in urine could provide interesting data on the involvement of the cleaved form of OPN and lupus nephritis induced by pristane.

Future studies exploring OPN as a marker for NPSLE should aim to confirm OPN’s role in generating specific neurotoxic autoantibodies, such as anti-N-methyl-D-aspartate receptor (anti-NMDA antibodies), through microglia activation. By establishing a potential link between circulating levels of OPN, its expression in microglia, and anti-NMDA production with neuropsychiatric manifestations in lupus models, this research could significantly enhance the accuracy of diagnosis and the potential for early treatment initiation in patients with NPSLE. Ultimately, this offers hope for improved patient outcomes, highlighting the positive impact of our study.

## 4. Materials and Methods

### 4.1. Animals

Female *BALB/c* mice aged 8–12 weeks old were housed in the animal facility of the University Center of Biological and Agricultural Sciences of the University of Guadalajara under the following conditions: 2–4 animals in clear cages (7.6 × 11.6 × 4.8 inches), a controlled temperature room at 22 ± 1 °C, a humidity of 40–60%, a positive laminar flow, 12 h of light/dark cycles, and the mice were fed *ad libitum* with norm caloric diet (SAFE^®^ A30) and purified water.

The protocol was approved by the Committee of Research, Ethics, and Biosecurity of the University Center of Health Sciences belonging to the University of Guadalajara (Protocol number CI-05622). All experimental procedures complied with the rules for animal research in health matters (NOM 0062-ZOO-1999 and NOM-033-ZOO-1995).

### 4.2. PIL Mice Model

Pristane (2,6,10,14 tetramethylpentadecane) is an isoprenoid alkane employed to induce a broad spectrum of lupus-specific autoantibodies in *BALB/c* mice. A total of 76 female *BALB/c* mice aged 8–12 weeks old were divided into three groups: pristane (P; *n* = 30), pristane plus lipopolysaccharide (P plus LPS; *n* = 32), and control (C; *n* = 14) groups. The induction of lupus in the P and P + LPS groups was conducted by a single intraperitoneal (i.p.) injection of 0.5 mL of pristane (Sigma-Aldrich^TM^, Saint Louis, MO, USA). The C group received a single i.p. injection of 0.5 mL of NaCl 0.9%.

### 4.3. Blood–Brain Barrier Disruption with LPS in PIL Mice

To induce BBB permeabilization, LPS was applied to an experimental group of mice previously treated with P. The P + LPS group consisted of female *BALB/c* mice who received a single i.p. injection of LPS from *E. coli* O55:B5 (Sigma-Aldrich^TM^, Saint Louis, MO, USA) in a dose of 3 mg/Kg diluted in NaCl 0.9% 16 weeks post-pristane administration.

### 4.4. Barnes Maze Test

We employed the Barnes maze (BM) test to evaluate rodents’ spatial memory and learning processes. The BM apparatus consists of a circular platform with holes in its perimeter, where only one hole leads to a dark escape chamber (escape hole). The characteristics and specifications of the platform used in our protocol were described previously [18].

Experimental animals started the BM test 7 weeks after LPS administration, and the behavioral procedures consisted of the following phases: habituation, acquisition, short-term memory (STM), sonda, long-term memory (LTM), and reverse tests.

*Habituation*: This initial phase tested the mice’s physical/visual recognition of the platform and their adaptation to the environment without aversive elements (no light, no sound).

*Acquisition*: This phase lasted three days, during which the repeated actions aimed to develop short-term memory (STM) and visuospatial memory and consolidate the process in long-term memory (LTM). During the development of each test, aversive elements (light and sound) were used for the study groups.

*STM*: This phase was performed after 3 days of acquisition and consisted of two tests (T1 and T2), in which the behavior of the mouse in the maze was analyzed under aversive stimuli such as sound at 95 decibels. The test had a maximum time of 180 s, which ended when the mouse entered and remained in the escape hole, taking this time as the latency of the test. The parameters that were considered in the Barnes test were the following:

- Duration per quadrant (seconds),
- Distance per quadrant (centimeters),
- Total distance traveled in the arena (centimeters),
- First appearance latency (the time it took for the mouse first to enter some quadrant of interest selected by the investigator; seconds),
- Escape hole entry latency (seconds).

*Sonda*: This phase was performed immediately after the conclusion of the STM and was used to analyze the mouse’s spatial navigation. It consisted of occluding the escape orifice so that the mouse recognized the correct location and oriented itself by signaling. This test lasted 90 s and analyzed the time the mouse remained over the escape orifice.

*LTM*: This phase was performed 72 h after the STM test to evaluate retention and long-term memory. During this time, the animal was not manipulated except for feeding. This test was performed under the same conditions as the STM test (sound, light, temperature, and time 180 s), being only one test.

*Reverse*: This phase was the last in the chronological order of the BM and was performed after the LTM test to analyze the problem-solving ability of the mice. The escape hole was changed over a range of 180 degrees relative to the previous position. It lasted 180 s and ended when the mouse found the escape hole and took shelter.

The parameters considered in the cognitive-behavioral assessment were as follows:

- Learning development (acquisition)
- Consolidating working memory (STM and LTM).
- Visuospatial memory consolidation (sonda)
- Cognitive reinforcement (reverse)

**Figure 7:**
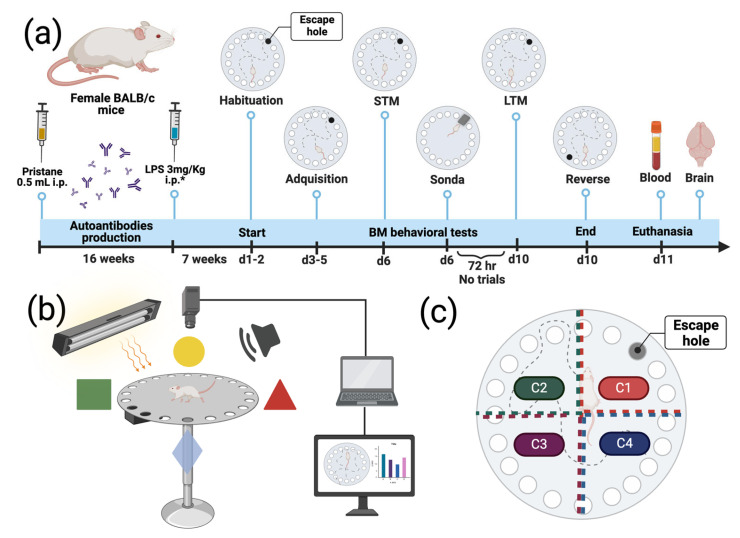
Schematic representation of the PIL mice model and behavioral assessment. (**a**) Timeline for the PIL mice model showing pristane and LPS application, BM behavioral assessment, and the collection of biological samples. (**b**) BM test procedure in which the elevated platform, the aversive elements, and the recording of videos for subsequent visual and software analysis are displayed. (**c**) Diagram of the platform arena divided into quadrants and interest zones for software analysis. * LPS application via i.p. was performed on the P plus LPS group. Abbreviatures: i.p. = intraperitoneal; mL = milliliters; mg = micrograms; Kg = kilogram; STM = short-term memory; LTM = long-term memory; BM = Barnes maze; d = day; hr = hour; C = quadrants. Created with Biorender. Agreement number XM27HGM1HF.

Figure 7 illustrates the study’s timeline, which includes the phases of behavioral assessment, the platform, the use of aversive elements, and the sand diagram for software evaluation.

Automated tracking was performed using the EthoVision^TM^ software 3.1.16 software (Noldus Information Technology) [31]. Table 1 describes the variables used in the evaluation protocol.

### 4.5. Quantification of Anti-Sm Antibodies

Once the BM test was finished, we obtained plasma from the whole blood of the tail vein and stored it at −20 °C. Subsequently, we performed the enzyme-linked immunosorbent assay (ELISA) using a 1:2 dilution to detect anti-Sm antibodies. Anti-Sm antibodies are directed against Sm antigens, considered specific and pathognomonic of SLE, and are part of the spectrum of autoantibodies produced in the *BALB/c* PIL mice model.

The quantitative kit Mouse Anti-Sm Ig’s total/A+G+M (Alpha Diagnostic International^TM^, San Antonio, TX, USA) was employed to quantify anti-Sm antibodies in plasma.

### 4.6. Quantification of OPN FL and OPN N-Half

For the determination of plasma OPN FL titers, we employed the Mouse Osteopontin Elisa Kit (Sigma St Louis, MO, USA). The determination of plasma OPN N-half levels was performed with the Osteopontin N-Half Mouse Elisa Kit (IBL^TM^, Minneapolis, MN, USA).

### 4.7. RNA Isolation and Spp1 Quantification by qRT-PCR Analysis

We performed the following procedures for quantifying OPN messenger RNA encoded by the *Spp1* gene in mouse (*Mus musculus*) brain tissue. The experimental animals were euthanized by CO_2_ inhalation, and the brain was removed after craniotomy surgery to obtain lysates for total RNA isolation according to the manufacturer’s procedure of the GF-1 Total RNA extraction kit (Vivantis Technologies^TM^, Selangor Darul Ehsan, Malaysia). Complementary DNA synthesis (cDNA) was performed with 5 μg of each total RNA sample using a reaction size of 20 μL with oligo (dT) primer (100 ng/μL), RNase-free, DEPC-treated water, and the Moloney Murine Leukemia Virus Reverse Transcriptase (M-MLV RT) kit (Thermo Fisher Scientific^TM^, Waltham, MA, USA), these were and stored at −20 °C until use for expression analysis. The TaqMan Fast Advanced Master Mix (Thermo Fisher Scientific^TM^, Waltham, MA, USA) and TaqMan gene expression assay (Thermo Fisher Scientific^TM^, Waltham, MA, USA) for *Spp1* (Mm00436767_m1) and Rps28 (Mm04203728_gH) were used to quantify *Spp1*. The 2^−ΔΔCT^ method was employed to assess fold changes in gene expression.

### 4.8. Statistical Analysis

The Kolmogorov–Smirnov test was used to determine the distribution of the data. Statistical comparisons were conducted with a one-way ANOVA and the Kruskal–Wallis test, as applicable. Values are presented as the mean and standard error of the mean (±SEM). Spearman’s correlation coefficients were also calculated. All data were analyzed using SPSS v22.0 (SPSS Inc., Chicago, IL, USA) and GraphPad Prism v 9.00 (GraphPad Software, La Jolla, CA, USA). *p* < 0.05 was considered statistically significant.

## 5. Conclusions

From the findings obtained in this study, we determined that the P-treated group presented alterations in visuospatial executive memory processes, cognitive reinforcement, and search attitude. These findings may be related to the association of anti-Sm titers with OPN FL at the systemic level and with the *Spp1* overexpression in the CNS.

Concerning the cleaved form of OPN, known as OPN-N half, this was found in lower levels in circulation compared to OPN FL; however, although no significance was found, the cleaved form of OPN was present in a higher proportion in anti-Sm-positive mice and may be related to LN in this model.

While the biological significance of the association of anti-Sm antibodies and OPN is yet to be fully understood, our findings suggest that OPN could be a crucial molecule in developing detrimental neurological conditions in the PIL mouse model. This potential impact on our understanding of neurological conditions underscores the need for further research in this area.

## Figures and Tables

**Table 1:** Variables measured with EthoVision^TM^ for BM task.

Variable	Description	Measurement	Unit
Total distance in the arena	Distance traveled in the arena to reach the escape area.	Total	cm
Total distance within the interest area	Distance traveled in the escape hole area.	Total	cm
First appearance latency	Time spent for the first time recognition of the escape hole.	Duration	s
Permanency in interest area	Time spent in the quadrant housing the escape hole (target quadrant).	Duration	s

Abbreviations: BM = Barnes maze; cm = centimeters; s = seconds.

## Data Availability

Data are contained within the article.
